# Perception of the Image of a Child and Oneself in the Role of a Mother by Women Parenting Disabled Children

**DOI:** 10.5539/gjhs.v7n5p326

**Published:** 2015-03-30

**Authors:** Svetlana Evgenyevna Inevatkina

**Affiliations:** 1 Mordovian State Pedagogical Institute named after M.E. Evseviev, Russia, Mordovia, 430007, Saransk, Studencheskaya str., 11 a., Russia

**Keywords:** children with disabilities, parent-child interaction, the image of a child, the image of oneself in the role of a mother

## Abstract

The article discusses the role of the parent-child interaction in the development of a young child with disabilities. It mentions possible distortions of the said interaction. In addition, the submitted material contains the results of an empirical study on the structure and content of the image of a child and perception of oneself in the role of a mother by women parenting children with disabilities.

## 1. Introduction

To date, the main role of the parent-child interaction in the development of a child has been proven many times. The influence of this factor is enhanced when it comes to the situation of parenting young children with disabilities. The interaction of the dyad “mother-child” holds a special place. For example, D.V. Winnicott in his publications noted that the child is a part of the relationship and cannot exist alone (Winnicott, 2004).

Domestic and foreign researchers note that the interaction with the mother or other important adults is the basis of development of all activities of a young child with disabilities and his/her relations with the world, but distortions of the parent-child relationships lead to emotional and behavioral disorders, disturbances of intellectual, motor, mental, and psychosomatic development of the child (Ainswort & Bowlby, 1991; Berne, 2010; Winnicott, 2004; Vygotsky, 2006; Leontyev, 2005; Lisin, 2009).

Analysis of literature gives reasons to make the assumption that a favorable change in the parental attitude and behavior will cause changes in the behavior and reactions of the child, and generally the parent-child interaction. In turn, researchers note that the parental behavior related to the interaction with the child is based on certain psychological education determining its peculiar features. For example, in the works of Russian scientists, it is said about the “parental position” (Spivakovskaya, 1989); E.B. Ayvazyan and E. I. Zakharova talked about the internal maternal position (Ayvazyan, 2008; Zakharov, 1997); western works often use the term “devotion of a mother to her child” (Leifer, 1977; Mercer, 2004).

The theoretical construct “internal maternal position” (IMP) was taken as the model for this study, which construct is developed on the basis of the cultural-historical approach to the development of the human psyche (Zakharova, 1997; Ayvazyan, 2008).

The internal maternal position can be understood as a form of reflection, adoption, and assimilation by a woman of her social position of the mother, which has the functions of planning and regulation of her own activities and communication. E. B. Ayvazyan formulated in a publication the assumption about the IMP structure as a hierarchically organized system of phenomena of the semantic, cognitive, and emotional nature. It built on the basis of the personalized sense of motherhood the images of oneself as a mother, a child, as well as relationship with him, which gradually becomes associated with certain expectations and percepts. In addition, based on it, the parental attitude and parenting style are formed. The sense of motherhood and the subjective value of a child in the mother’s life determine also the content of emotions included in the attitude to him (Ayvazyan, 2008).

That is, the image of oneself in the role of a mother and the image of a child are the components of the internal maternal position, which organize the perception and understanding of the child and oneself, the feelings towards the child and oneself, and the actual behavior within the interaction.

We selected the situation of giving birth to and upbringing a child with the Down syndrome as the experimental model in this study. Our choice is determined by the fact that this syndrome is one of the most common genetic abnormalities: its frequency among newborns is 1:600900 on the average and in the case of the mother over 45 years old 1:32. This syndrome is based on the presence of an extra chromosome in the 21 pairs, and its manifestations include specific physical appearance and a number of mental and physical disorders.

Despite the proliferation of the Down syndrome among children, there are a few publications dedicated to the child-parent relationship in their families, and the existing ones are mainly aimed at the analysis of the peculiar features of the interaction of a mother and a child (Inevatkina, 2011). For example, a number of scientists in their publications have pointed out that the peculiar features of the development of a child with the Down syndrome (possible physical problems, lower cognitive activity, weak response to communication, delayed development of the smile and the “eye to eye” contact, etc.) can hinder the interaction of a mother with her child and the formation of her devotion (Berger & Cunningham, 1981; Emde & Brown, 1978; Emmanuel et al., 2008). In addition, the situation of close interaction of relative adults and a child with the Down syndrome may be partly complicated by the attitude of the society to the family with a child that has developmental disabilities noticeable to others, which attitude is often contradictory and “loaded” with bias (Goffman, 1963; Malofeev, 2009; Nelson, 2003; Saurel-Cubizolles et al., 2007). Besides, the behavior of the mother can be determined by her “psychological health” (Berger & Cunningham, 1983; Edge & Rogers, 2005; Emmanuel et al., 2010) or the psychological safety of the subject (Vardanyan & Ruskina, 2013).

Thus, the goal of our research is to study the structure and content of the IMP of mothers who parent young children with the Down syndrome, in particular, to study the image of oneself as the mother and the image of a child.

## 2. Methodology

The empirical study involved 139 pairs of mothers and young children, including: the experimental group–47 pairs “mother–child with the Down syndrome” (the DS group); the control group–92 pairs “mother–a normally developing child” (the NN group). The control and experimental groups were equated by age and socio-demographic characteristics of mothers–education, employment at the time of the study, marital status.

In order to study the image of oneself as a mother and the child image of women parenting young children with the Down syndrome, we used as the main technique the projective method “Incomplete sentences” (IS) designed by Arina and Ayvazyan. As a tool allowing to “bypass” the protective mechanisms and the factor of social desirability of the response, the authors of the method used the technique of controlled projection suggested by V. V. Stolin (1983). The method resides in comparing the responses attributed to the character synthesized according to the data of the MMPI test and the responses, in which own position is “directly” presented. This comparison allows exploring both the unconscious and declared values. The test consists of 27 unfinished sentences that make up 9 scales united in 3 blocks: 1) the content of the semantic sphere (the scales “Meaning of life”, “Meaning of the family”, “Meaning of marriage”, “Meaning of motherhood”; totally 12 IS); 2) the emotional and value-related attitude to the child (the scales “Attitude to the child now”, “Expectations about the future of the child”; 6 IS); 3) the emotional and value-related attitude toward oneself (the scale “Attitude to oneself as a mother”, “Evaluation of oneself through the eyes of the husband”, “Evaluation of oneself through the eyes of children”; 9 IS).

The answers of the tested persons were subjected to the content analysis, i.e. all the answers depending on their content were broken up into certain categories, which were subsequently divided by the topics “Image of oneself as a mother” and “Image of the child”. In the context of the topic “Image of oneself as a mother”, we identified: the value-related social roles of the tested persons (for example, the role of a mother, the role of a wife, the role of a hostess, the role of a leader); the value-related functions (e.g., the function of caring, understanding, dedication); the limit values (for example, happiness, health, welfare); the value-related characteristics (e.g., activity, gentleness, kindness, optimism); the semantic aspect of the emotional and value-related attitude to the child, i.e. description by the mother of her attitude and feelings to the child (e.g., love, tenderness, kindness, joy, anxiety, anger). The topic “Image of the child” included value-related characteristics of the child (e.g., his/her activity, success, health, and personal well-being). Further processing of the results was performed by means of the frequency, factor, and correlation analysis.

## 3. Results

The results of empirical studies allowed describing the specificity of the image of the child in the DS group compared with the NN group.

The first discovered phenomenon concerns the results of the analysis of the hierarchy of the child’s value-related characteristics. It was found, that the importance of the social achievements of the child was in the first place in both groups. That is the birth of a child with the Down syndrome does not change the internal orientation of the mother to the child’s social success. But in the DS group, unlike the NN group, the priority in the hierarchy of the value-related expectations along with the social solvency was given to the characteristics of the subjective well-being of the child – his/her personal happiness, satisfaction, and internal comfort.

The second phenomenon concerns the fact that for the mothers of children with the Down syndrome (as opposed to the tested persons of the NN group), such a characteristic as “Obedience” is not significant (its frequency is only 8.5%, while the standard rate is 38%, p≤0.05). There are several explanations of this fact. Obedience in the system of value-related expectations can have several meanings. On the one hand, obedience is a “tool” of parenting, in accordance with the content of common social stereotypes–it is a mandatory prerequisite for “growing” the positive personal characteristics of the child and ensuring his/her social achievements. Decline in the importance of obedience in this semantic context may indicate, for example, that social success is perceived as inaccessible, and the need for obedience in its function as a way of achieving it disappears. However, this assumption contradicts to the high importance of social achievements in the hierarchy of the value-related characteristics of a child. Consequently, we can assume that mothers of children with the Down syndrome vary the idea of the methods to achieve social success, for example, they can treat personal well-being and comfort inside as a method of development of socially significant characteristics in the child.

On the other hand, obedience is a characteristic of the “comfort of the child for the parents”, his/her predictability and manageability. Consequently, the decline in the importance of obedience in the DS group may indicate recognition of the child’s own will (“he has the right to be uncomfortable for the parents”). Reasons for this change in the system of values are different as well: either the parents of children with the Down syndrome, for whatever reasons, are more democratic, or they are more lenient because the child’s defect causes them not to require the child to behave in a “standard” manner and even makes them to “give up”.

In addition, an “obedient child” is an evidence of the success of a woman in her maternal role, the effectiveness of her parental strategies or even a sign of her great contribution to the parenting of the child. Decline in the value of obedience in this context may indicate that for mothers of children with the Down syndrome, the maternal success seems unattainable, or its criteria change. Thus, in a situation of raising a child with the Down syndrome, the child image and the system of value-related expectations, addressed to him, are greatly transformed – not only the compliance of the child with the social requirements become important, but also meeting his/her internal, psychological needs. We can say that there is a change in the position of the ’children and parents’ relations – from the “subject-object” towards the “subject-subject” relations. Apparently, this change is reflected in the transformation of concepts of parenting strategies.

We can assume that it is the result of activity of experts engaged in the socio-psychological and pedagogical support to families. In our study, mostly those families participated, which were receiving assistance at the specialized Center of Early Intervention for Children with the Down Syndrome “Downside Up” (Moscow) and the Center for Special Education of the Samara region. The activities of these organizations are aimed at organizing successful inclusion in the social life of children with the Down syndrome and their families.

The obtained results give reasons to assume that the impact of these services will be reflected in the value-related expectations addressed to oneself as a mother. To test this hypothesis, we undertook a study of the specificity of the image of oneself as a mother in the DS group compared with the NN group.

The first phenomenon, which manifests the specificity of the DS group, was found in the study of the structure of values related to self-fulfillment.

The specificity of the group of women parenting a child with the Down syndrome is that it lacks high frequency combinations of categories – the results of this group showed a smaller number of correlations. Consequently, the value-related space of this group is highly individual.

**Figure 1:**
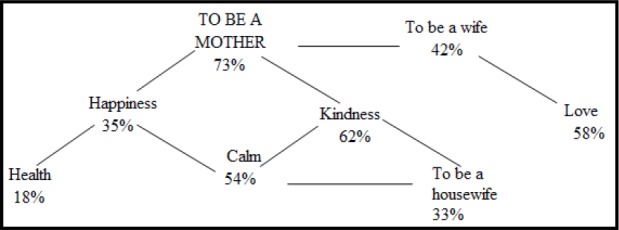
Statistically significant correlations with the category “To be a mother” in the NN group

The category “Being a mother” in this group does not have a single statistically significant correlation, whereas in the NN group, this category became the center of the whole pattern of categories (Figure 1).

Positive correlations found in the results of the NN group give reasons to state that if a woman is oriented to fulfillment of herself in the role of a mother, then she finds value in the roles of a hostess and a wife, showing care to her husband, and the opportunity to rely on a loved one, as well as such value-related characteristics as kindness, peace, happiness, care, love.

Thus, women parenting children with the Down syndrome have no self-fulfillment stereotype common for the whole group.

**Figure 2:**
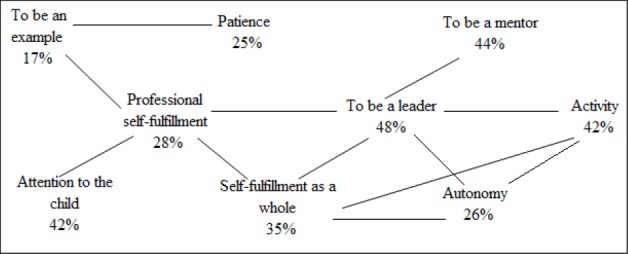
Statistically significant correlations with the category “Professional self-fulfillment” in the NN group

The same situation is observed at the analysis of the values associated with professional self-fulfillment. The results of the DS Group show no statistically significant correlations with the category “Professional self-fulfillment”. As for the tested persons of the NN group, professional self-fulfillment is a component of the value-related pattern, which also includes the values of the leadership, mentoring, and authoritative position, success, attention spared to the child, as well as a number of characteristics, such as honesty, independence, and active position (Figure 2).

Thus, for the tested persons in the NN group, the professional self-fulfillment is a part of the lifestyle, which along with the “extra-family” roles has the roles addressed directly to the child (to be an example, to be a mentor), which provides a balance of self-fulfillment in the family and at work.

The tested persons in the DS group again have no stereotype of professional fulfillment common for the group. This fact is also complemented by the results of the frequency analysis, which showed that the category “Professional self-fulfillment” is used less frequently by the tested persons in the DS group than by the tested persons in the NN group (p<=0.05).

## 4. Discussion

Accordingly, the obtained data allow us to see that in the NN group there is a common for the group vision of self-fulfillment in the maternal role and profession. These two definitely key positions in self-fulfillment of a woman are accompanied by certain expectations and perceptions of the characteristics and functions associated with these roles. In the group of mothers parenting children with the Down syndrome, there is not only no standard method (the method observed in the NN group) of determining the content of these social roles, but also no method that would be common for the group. This fact allows us to formulate two hypotheses to explain it. Firstly, it is possible that in the DS group these “standard” strategies of determining the social roles are destroyed after the birth of the special child. Secondly, it is possible that in the NN group, they are formed by this time, and in the DS group, their formation is delayed. To verify the hypothesis described above, we compared our data with the data obtained in the study of value-related social roles at the stage of pregnancy. The results of the study conducted by E. B. Ayvazyan (2005) give reasons to reject the second explanation: Even at the stage of pregnancy (pregnancy without problems), the sufficiently formed, common for the group methods of describing self-fulfillment in the maternal and professional roles are discovered, similar to those we identified in the control group. Hence, the birth of a child with the Down syndrome makes the usual stereotypical ways of self-fulfillment in maternal and professional roles inaccessible, forcing women to seek their own solution, redefine both the content of these roles and the ways of their combination.

The second phenomenon, discovered when comparing the DS and NN groups concerns the results of the analysis of differences between direct answers (when the tested person answered “for herself”) and indirect ones (when the tested person replied for a portrait drawn by the results of her MMPI test). The content of these differences allows estimating how the value-related orientations are converted at their comprehension and direct presentation.

In both groups, the transformation of value-related orientations resides in downplaying the leadership aspirations. Probably, this is the direction of the substantial transformation of value-related orientations, in which the adherence to the socially approved mindset is reflected, according to which a woman with a young child should not strive for self-fulfillment in active, “leading” social roles.

However, in the DS group, the effect of the factor of social desirability of a response is expressed to a much lesser extent. In the NN group on the declared level, firstly, the importance of family roles – the mother, the wife, and the hostess – is exaggerated (the frequency of the corresponding categories for direct responses is significantly higher than for indirect ones). Secondly, in this group, the significance of the value-related functions, such as attention paid to the husband, care, love, understanding, and commitment, is exaggerated. Thirdly, the importance of “passive” personal characteristics – calm and patience – is exaggerated.

This does not happen in the DS group, i.e. the tested persons in the DS group do not try to seem more humble and “family-centered” than they actually are. Moreover, for the mothers in the DS group, such personal characteristics as optimism and activity turn out to be more significant than in the “normal” group (these categories are found in the DS group significantly more often in both direct and indirect responses), and the value of these characteristics is not “hidden” as in the “normal” group.

Perhaps, it is explained by the fact that life problems associated with the birth of a child with the Down syndrome do not leave psychological stamina to try to comply with social expectations. Optimism and activity in this case are a certain mechanism to cope with the difficulties, so the desire to possess these characteristic can be shown directly.

However, the following explanation seems more appropriate: the dependence of the tested persons on social expectations is less pronounced, because there is no intelligible system of social expectations addressed to the mother of a special child. Moreover, the lack of value references formulated by the society may in part determine the “dithering” of internal strategies of self-fulfillment, which we identified during the analysis of value references.

Analysis of the differences between direct and indirect answers in the DS group allows detecting the influence of a particular stereotype that exists in the society and affects mothers of a “special child”.

For example, with the direct description of the limit values in the NN group, the importance of happiness is exaggerated, and at direct description of their feelings for the child, the tested persons in the NN group exaggerate the feelings of love, tenderness, and joy (the frequency of these categories at direct answers significantly increases, p<=0.05, compared to the indirect ones). This does not happen in the DS group; in addition, indirect answers in this group express more love and tender feelings for the child than in the NN group.

The explanation regarding the smaller effect of the factor of social desirability of the answer suggested above is no longer enough. Perhaps, the social stereotype, which does not allow recognizing the positive feelings in the situation of giving birth to a child with a genetic abnormality, even if they are present, begins to take effect.

Thus, the image of oneself in the role of a mother also has certain specificity. The “standard” ideal of self-fulfillment in the role of a mother, which had been forming during pregnancy, or even before its occurrence, ceases to function. With the birth of a “special” child, the model of motherhood created by the time of delivery is destroyed or rejected, and in the first three years of the life of the child, a new model common for this category of women does not form. We can assume that this is due to the lack of a coherent system of social expectations, addressed to the mother of a special child, as well as by virtue of the contradictions that exist between public expectations and informing by the public services. Firstly, the services call mothers for active and responsible position, and the society demands obedience and dependence on the family, specialists, and circumstances. Secondly, the services call mothers for emotional contact with the child, saturated with joy and optimism, and the society provides the mother of a special child with the stereotype of “plight”, which cannot be associated with positive experiences. It is obvious that the lack of a consistent view of the necessary maternal characteristics and an adequate maternal behavior in the social space makes women to need a new axiological self-determination, and one cannot sometimes solve this problem without assistance.

## 5. Conclusion

The interaction of the mother and the child, the dyad “mother-child with development disorders”, and even broader – the “family raising a disabled child”, become the object of early intervention in special education. Accordingly, the very subject of the research in the special pedagogy and special psychology changes – it is now the interaction of the mother and the child, as well as the factors determining its peculiar features, in particular, the psychological singularities of the mother, which govern her behavior within the interaction.

Thus, the functions of early intervention services should include not only forming a “positive image of a child with the Down syndrome”, but also changing the system of value-related orientations of the parents parenting a “special child”. To solve this problem, it is necessary not only to inform, educate, and provide deep psychotherapeutic work, but also to change the social stereotypes in the society – to create a positive model of a “special” child’s mother and his/her family as a whole.
